# Awareness and practice of birth preparedness and complication readiness among pregnant women in the Bamenda Health District, Cameroon

**DOI:** 10.1186/s12884-019-2511-4

**Published:** 2019-10-22

**Authors:** Yunga Patience Ijang, Samuel Nambile Nambile Cumber, Claude Ngwayu Nkfusai, Mbinkar Adeline Venyuy, Fala Bede, Pierre Marie Tebeu

**Affiliations:** 1 Department of Public Health, School of Health Sciences, Catholic University of Central Africa, Box 1110, Yaoundé, Cameroon; 2 Institute of Medicine, Department of Public Health and Community Medicine (EPSO), University of Gothenburg, Box 414, 405 30 Gothenburg, SE Sweden; 3 Faculty of Health Sciences, University of the Free State, Bloemfontein, 33 South Africa; 4 School of Health Systems and Public Health, Faculty of Health Sciences, University of Pretoria Private Bag X323, Gezina, Pretoria, 0001 South Africa; 5 Cameroon Baptist Convention Health Services (CBCHS), Yaoundé, Cameroon; 6 Department of Microbiology and Parasitology, Faculty of Science, University of Buea, Buea, Cameroon

**Keywords:** Birth preparedness and complication readiness, Health district

## Abstract

**Background:**

Birth preparedness and complication readiness has as goal to reduce maternal and neonatal mortality. This concept developed by the organizations of the United Nations permits pregnant women and their families seek health care without delay in case of obstetric complications and delivery. Though its benefits have been proven in several countries, little is known of this in Cameroon and specifically in the North West Region. Therefore, the intention of the study was to assess the awareness and practice of birth preparedness and complication readiness in this health district.

**Methods:**

This was a facility-based cross sectional study carried out in the Bamenda health district of the North West Region, Cameroon. Three hundred forty-five pregnant women of ≥32 weeks gestational age seen at the antenatal consultation units were recruited. The dependent variable was birth preparedness and complication readiness while the independent variables were the socio-demographic and reproductive health characteristics. Data collected was analyzed with SPSS and Microsoft excel. Frequency distributions were used to determine the awareness and practice of birth preparedness and complication readiness.

**Results:**

Of the 345 pregnant women included in this study, 159(46.1%) were aware of birth preparedness and complication readiness. The practice of birth preparedness and complication readiness was unsatisfactory as only 65(18.8%) were considered prepared.

**Conclusion:**

Education and counselling on birth preparedness and complication readiness is not made available to the pregnant women resulting in poor knowledge. Thus, reflected in the low practice of preparation for birth and its complication observed.

## Background

Maternal mortality is still a major public health problem although globally there has been a decrease [1]. Unfortunately, Cameroon has its maternal mortality ratio (MMR) increased from 430 per 100 000 live births in 1991 to 782 in 201 1[2]. Birth preparedness and complication readiness (BPCR) among other strategies developed by the safe motherhood programme of the United Nations was recognized as a key component in the reduction of maternal and neonatal mortality [3].

Birth preparedness and complication readiness is a comprehensive package to promote timely access to skilled maternal health services. It permits pregnant women and their families seek health care without delay in case of obstetric complications and delivery [3, 4]. Birth preparedness and complication readiness (BPCR) is the process of planning for normal birth and anticipating the actions needed in case of an emergency [3]. BPCR is an essential component of the focused antenatal consultation (FANC) adopted in Cameroon by the Ministry of Public Health to combat the two main delays out of three that are known to be associated with most maternal deaths [5]. It is evident that most of the complications that lead to maternal deaths if treated on time will greatly reduce maternal mortality. The aim of BPCR is to permit pregnant women and their families to overcome the delays that often lead to fatal outcomes due to absence of timely care [3]. BPCR consists of the pregnant woman and her family making active preparation and decision making on identifying a health facility and a skilled birth attendant, saving funds for delivery, emergency and transportation, arranging for mode of transportation, identifying compatible blood donors, arranging necessary article, identifying birth companion and having knowledge on danger signs [2, 3]. Birth preparedness and complication readiness, a strategy to fight this dilemma of maternal and neonatal mortality, is yet to gain its root as a pillar of the focused antenatal consultation. Despite the fact that it is considered as a simple and a practicable means of reducing maternal and neonatal mortality, it is not widely implemented by women and their families as evidenced by maternal deaths still occurring due to delays. Therefore, the objective of this study to provide analysis on the awareness and practice of BPCR among pregnant women in the Bamenda Health District. The results of this study can provide suggestions which may be beneficial in the reduction of maternal morbidity and mortality.

## Method

### Study design

This was a facility-based cross-sectional study conducted in the Bamenda Health District (BHD) of the North West Region of Cameroon.

### Study duration

This study was conducted from February to December, 2016.

### Study population

The target population was pregnant women who were at 32 weeks of pregnancy and above and attending ANC at chosen government health facilities in the Bamenda Health District.

### Criteria of inclusion

- Willing and able to give informed consent.
- Women with ≥32 weeks of pregnancy and attending ANC at the chosen health facilities.
- At 32 weeks of pregnancy, the women are expected to have made preparations for birth/complications.

### Criteria of exclusion

- Women attending ANC visit for the first time

### Sampling method

The non-probabilistic convenience sampling was employed to select the study population. Therefore, any pregnant woman who was eligible and willing to participate was included. The sample size was obtained using the Schlessman formula.

$$ \mathrm{N}=2\mathrm{P}\ \left(1-\mathrm{P}\right)\ \left(\mathrm{Z}\upalpha +\mathrm{Z}\upbeta \right)\ 2/{\left(\mathrm{Po}-\mathrm{P}1\right)}^2 $$

From the above formula, the sample size was 345 women.

### Variables

#### Dependent variable

The dependent variable was birth preparedness and complication readiness. The women were grouped as “prepared” and “unprepared”. Women were considered “prepared” on these seven aspects of BPCR (identify health facility, saved funds for birth/complications, means of transportation in birth/emergency, identify blood donors, packed necessary materials for birth, identified decision-maker and birth companion) while those who had done less than these seven aspects were “unprepared”.

#### Independent variables

The independent variables to be associated with the dependent variable are:

- Socio-demographic factors (age, level of education, marital status, religion, residence, income).

- Reproductive health factors (parity, gestational age at first ANC, and number of ANC visits).

### Data collection tools/technique

The tool for data collection was a structured questionnaire filled by the investigator. The questionnaire was adopted from the survey tools developed by JHPIEGO Maternal Neonatal Health program. A pre-test of the questionnaire was done at the Bamenda Regional Hospital to ensure its feasibility to respond to the objectives of the study.

Two state registered nurses were trained and helped in the data collection. The technique of the data collection was through a face to face interview with the participant to elicit responses if eligibility for the study within the period of data collection. Although the original questionnaires were in English language, pidgin English had to be used for those who could not understand English.

### Data processing and analysis

Data collected was coded and entered into Microsoft Excel. It was then transferred and analyzed using SPSS version 20 software. The prevalence of awareness and practice of BPCR were determined from simple frequency distribution. To be considered “prepared”. The woman should have taken seven steps of BPCP plan. Pearson’s chi-square was done to test for association between the dependent variable BPCR and the independent variables. Factors that were found to have p-values below 0.2 were analyzed on bivariate and multivariate logistic regression at 95% confidence interval and p<0.05 were done to identify factors that favor with birth preparedness and complication readiness.

### Operational definitions

Birth preparedness and complication readiness: a woman was considered to the prepared for birth/complications if she identified seven of the components of the BPCR items.

Awareness of BPCR: was considered when the woman had heard the term birth preparedness or delivery plan which may be commonly used in our context.

Complications in pregnancy: involves health threats to the unborn baby and the mother.

Knowledge of danger signs in pregnancy: a woman was knowledgeable if she could spontaneously give at least any three danger signs.

### Ethical consideration

Ethical approval for the study was obtained from the Institutional Research Ethics Committee for Human Health (CIERSH) at the School of Health Sciences of the Catholic University of Central Africa (reference N° 2016/0431/CEIRSH/ESS/MSR). Administrative clearance was obtained from the Delegation of Regional Health Bamenda.

A written consent form was signed by each participation willing to take part after going through the participant’s information sheet. Parental consent was sought for minors under the age of 16.

## Results

A total of 345 pregnant women were included in the interview. They comprised of pregnant women with gestational age ≥ 32weeks. The modal age range was 25 – 34 years with the least aged being 15 years and the most 44 years.

**Fig. 1:**
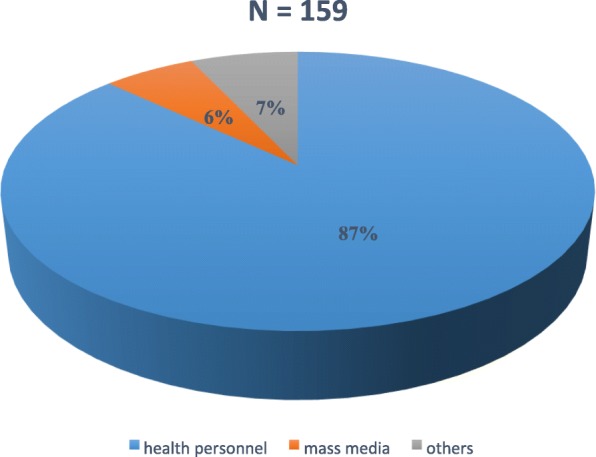
Source of information on birth preparedness and complication readiness 5

**Fig. 2:**
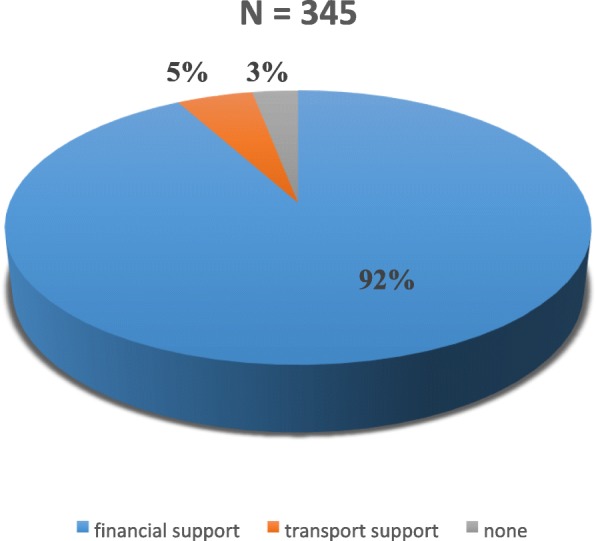
Knowledge on community support services to assist pregnant women

Of the 345 participants in this study, the proportion of pregnant women who had heard of the term birth preparedness was at 46.1%. Even though, only 46.1% acknowledged to have heard of the term birth preparedness, a majority of the pregnant women 308(89.3%) acknowledged to have received some kind of information on preparations to be made during pregnancy from the health workers (Fig. 1). However, only 96(27.8%) testified to this information being provided or followed up at each visit. The proportion of pregnant women with written birth plan was at 6.7%. One hundred and eighty-three (53%) women received information on danger signs in pregnancy, 298(86.4%) received on where to go in case of health problem, 283(82%) on where to give birth, 308(89.3%) on making arrangement for funds, 165(47.8%) were informed to make arrangements for transportation, 102(29.6%) on arrangement for blood donors, 54(15.7%) were asked to choose a desired health worker to deliver the baby, and 231(67%) were informed on signs of labor (Fig. 2). A mean proportion of 58.85% of information on BPCR were acknowledged to have been received by the participants in this study (Table 1). On the knowledge of the possibility of unforeseen health problems that can occur during pregnancy leading to the death of the woman, 305(88.4%) reported that it was possible while 40(11.6%) responded that it was impossible. The pregnant women were questioned on whether they knew any danger signs of pregnancy. Regarding the knowledge of dangers signs, 87.5% indicated that they knew some danger signs and gave examples of them while 12.2% of them did not know any danger signs in pregnancy. Vaginal bleeding was the most frequent mentioned danger sign (73.9%), severe abdominal pain (40.6%), high fever (21.2%), abnormal fetal movements (17.4%), leakage of amniotic fluid before labor (15.7%); difficult breathing (11.3%); swollen hands/face/feet (6.7%), convulsion (5.2%); blurred vision (0.9%), severe headaches (6.7%), severe weakness (6.1%), loss of consciousness (2.6%), and others including severe vomiting, foul-smelling discharge (2.0%) (Table 2).

**Table 1:** Information provided by health workers on birth preparedness and complication readiness

Information on birth preparedness and complication readiness	Total = 345	
	Yes (*n* = %)	No (*n* = %)
Heard of birth preparedness	159 (46.1)	186 (53.9)
Health worker discussed on preparations	308 (89.3)	37 (10.7)
Birth plan discussed at every visit	96 (27.8)	249 (72.2)
Written birth plan	23 (6.7)	322 (93.3)
Danger signs in pregnancy	183 (53.0)	162 (47.0)
Where to go in case of serious health problems	298 (86.4)	47 (13.6)
Where to give birth to your baby	283 (82.0)	62 (18.0)
Arrangements for funds	308 (89.3)	37 (10.7)
Arrangements for transportation	165 (47.8)	180 (52.2)
Arrangement for blood donors	102 (29.6)	243 (70.4)
Arrangement for the health worker to deliver your child	54 (15.7)	291 (84.3)
Signs of labour	231 (67.0)	114 (33.0)

**Table 2:** Respondents’ knowledge of danger signs in pregnancy

Danger signs	Number of responses *N* = 345	Percentage (%)
Vaginal bleeding	255	73.9
Swollen hands/face/feet	23	6.7
Convulsion	18	5.2
Fever	73	21.2
Difficult breathing	39	11.3
Severe abdominal pain	140	40.6
Blurred vision	3	0.9
Severe weakness	21	6.1
Severe headache	23	6.7
Absence of foetal movement	60	17.4
Loss of consciousness	9	2.6
Amniotic fluid leaks	54	15.7
Others (severe vomiting)	7	2.0

In the practice of BPCR, 287(83.2%) had saved money/ kept money aside for incurring cost of delivery and obstetric emergencies, 197(57.1%) had identified and made arrangement for means of transportation, 23(6.3%) had identified skilled birth attendant, and 79(22.9%) had identified blood donor. Taking at least seven steps was considered being well prepared. Accordingly, less than one fifth (18.8%) of the pregnant women in this study were considered well prepared for birth and complications (Table 3).

**Table 3:** Socio-demographic and reproductive health characteristics of the respondents according to birth preparedness and complication readiness

Elements of preparation	Number of respondents (*N* = 345)	Percentage (%)
Saved money for birth/complications	287	83.2
Identified mode of transport	197	57.1
Identified blood donors	79	22.9
Identified health facility	276	80.0
Identified skilled health worker	23	6.3
Identified birth companion	322	93.3
Identified decision maker	328	95.1
Packed necessary items for birth	245	71.0
Number of elements prepared		
1	1	0.3
2	11	3.2
3	24	7.0
4	80	23.2
5	85	24.6
6	79	22.9
7	46	13.3
8	19	5.5

In bivariate logistic regression analysis of the socio-demographic and reproductive health characteristics of the respondents, level of education, occupation, monthly income, number of ANC visits, and knowledge of danger signs in pregnancy were significantly associated with birth preparedness and complication readiness. Multivariate logistic regression showed monthly income (p = 0.013) and number of ANC visits (p = 0.013) to be significantly associated with birth preparedness and complication readiness (Table 4**).**

**Table 4:** Bivariate and multivariate analysis of socio-demographic and reproductive health characteristics influencing birth preparedness and complication readiness

Characteristics	Birth preparedness and complication readiness *N* = 345					
	Unprepared N = 280 n (%)	Prepared N = 65 n(%)	Bivariate OR (95% CI)	*p*-value	Multivariate OR (95% CI)	*p*-value
Age						
15–24	65 (23.2)	17 (26.1)	1^c^		1^c^	
25–34	173 (61.8)	38 (58.5)	7.43 (0.95, 58.40)	0.057*	5.10 (0.58, 45.12)	0.143
35–44	42 (15.0)	10 (15.4)	6.57 (0.52, 83.76)	0.147	7.42 (0.51,108.41)	0.143
Level of education						
Primary and below	101 (36.1)	9 (13.8)	1^c^		1^c^	
Secondary	132 (47.1)	30 (46.1)	2.55 (1.16, 5.61)	0.020*	2.1 (0.92, 4.8)	0.079
University	47 (16.8)	26 (40.0)	6.21 (2.70, 14.28)	0.001*	2.5 (0.95, 6.73)	0.064
Occupation						
No employment	101 (36.1)	22 (33.8)	1^c^		1^c^	
Gov’t employee	21 (7.5)	16 (24.6)	3.50 (1.58, 7.76)	0.002*	0.75 (0.18, 3.15)	0.699
Private/self-employee	158 (56.4)	27 (41.5)	0.79 (0.4, 1.45)	0.440	0.50 (0.24, 1.02)	0.056
Monthly income						
0–49,999	213 (76.1)	28 (43.1)	1^c^		1^c^	
50,000–99,999	64 (22.8)	33 (50.8)	3.29 (1.69, 6.40)	0.001*	2.94 (1.39, 6.25)	0.005*
100,000–300,000	3 (1.1)	4 (6.1)	5.95 (2.86, 12.38)	0.001*	3.0 (0.81, 11.68)	0.100
Residence						
Rural	100 (35.7)	17 (26.2)	1^c^		1^c^	
Urban	180 (64.3)	48 (73.8)	1.57 (0.86, 2.87)	0.145	1.63 (0.82, 3.22)	0.162
Number of ANC visits						
2–3	209 (74.6)	36 (55.4)	1^c^		1^c^	
4–9	71 (25.4)	29 (44.6)	2.37 (1.35, 4.14)	0.002*	2.16 (1.18, 3.90)	0.013*
Knowledge on danger signs						
0–2	198 (70.7)	36 (55.4)	1^c^		1^c^	
3–5	82 (29.3)	29 (44.6)	1.95 (1.12, 3.38)*	0.018*	1.39 (0.75, 2.58)	0.294

*significant values (*p* < 0.05)

## Discussion

Awareness of the term birth preparedness was found to be at 46.1% (159 respondents). This is similar to the 46.4% in Oromia region, Ethiopia [6] but lower than the 70.6% in South-eastern Nigeria [7]. The main source of information on birth preparedness was provided by health professionals (89.1%), similar to studies in Ethiopia [8, 9]. Many women (89.3%) acknowledged to have been provided with some information on preparations to be done, higher than the 68.3% reported by [4] in Ethiopia.

Information provided by the health workers included where to go in case of health problem or birth, arrangements of funds, arrangements of transportation. The results obtained were higher compared to those in Ethiopia and Ghana [4, 10]. Up to 70.4% of respondents were not informed on the need to identify blood donors which is high to that of 45.8% and 54.2% in Ghana and Kenya [10, 11]. Information received on the identification of SBA during delivery had the least proportion of 15.7%. The mean value for information received by pregnant women from health providers was at 58.85% which confirms that the awareness of the components of BPCR is low.

The proportion of women who had received information on danger signs was higher than the national proportion of the 2011CDHS and that of a study in the Buea Health District [2, 12] but lower than that of 77% in the North West Region of Cameroon [2] and 90.38% in Ghana [13]. There was low awareness of danger signs indicated in this findings, higher than in Mpwapwa, Tanzania, rural Uganda and others [14–16]. The finding is however lower than in Ghana and Ethiopia [17, 18]. The level of education is statistically associated with the knowledge in danger signs where women with university education were three times (OR=3.14; 95% CI: 1.67, 5.93) more likely to be knowledgeable than those with primary education and below, similar to [18] in Ethiopia. This study indicated a low proportion (18.8%) of pregnant women were well prepared for birth and obstetric complications. This is similar to a study in Wolayta zone, South Ethiopia [19] with 18.3% but lower than studies in Ethiopia, Uganda, and Nigeria [6, 15, 20, 21]. The difference in findings may be due to the differences in study population and the number of elements considered for preparedness. Majority of the women had saved money for birth/complications, similar to findings in [22–24].

The level education was statistically associated with BPCR on bivariate analysis in this study. Those in the University were six times more likely to prepare for birth or complications in pregnancy (OR = 6.21, 95% CI: 2.70, 14.28) compared to those with primary level and below. Similarities to this finding were seen in studies in Kenya and Nigeria [25]. Occupation was also associated with BPCR (OR = 3.50, 95% CI: 1.58, 7.76). In this study, women who were employed by the government were 3.5times more likely to prepare for birth and complications than those with no employment (students, housewives, farmers). This finding is quite similar to a study in Ethiopia [6]. Monthly income was a predictor of BPCR (AOR = 2.94, 95% CI: 1.39, 6.25). This is consistent with studies in Kenya, Nigeria, India and Uganda [10, 24, 26, 27]. An increase in the average income of an individual has a positive influence on the likelihood of preparing for birth and its complications. This relates to the three delays model where the first delay (decision to seek care) is related to socio-economic and cultural factors. These could be due to the fact that the economic status of the woman gives her the ability to make wise decision and payment on her own than their counterparts. The number of ANC visits is also predictor of birth preparedness (AOR = 2.16, 95% CI: 1.18, 3.90), similar to studies in Tanzania [16, 22] where women who attended at least four ANC visits were approximately two times more prepared than women who attended less. One of the basic assumption stipulated by the concept of BPCR is the fact that the knowledge of danger signs will lead to a greater preparation to minimize effects of complications in pregnancy and child birth. This is so by reducing the first two delays common in untimely care. In this study, women who had knowledge on obstetrics danger signs were more likely to be prepared for birth and its complication compared to those who did not know. The finding of this study showed that knowledge of danger signs is associated with BPCR (OR = 1.95, 95% CI 1.12, 3.38; p = 0.018). This is similar to studies in Nigeria, Ethiopia, India and Uganda [14, 26, 28, 29]. In this study those knowledgeable of danger signs were 1.96times more likely to be prepared than those without.

## Conclusion

The findings of the study revealed that the awareness of birth preparedness and complication was low 159 (46.1%). Majority of the pregnant women did not make preparations as required by the BPCR plan. Only 65(18.8%) were prepared according to this study. Insufficient information provided by the health providers and absence of community health workers in the communication of the message on birth preparedness and complication readiness could be attributed to the low status of birth preparedness and complication readiness among pregnant women in this health district.

This study provides an important contribution in the fight against maternal and neonatal mortality. Due to the importance of this subject, a qualitative study to understand why the sufficient preparations are not made can be done in this health district. To the problems identified in this study, the following proposed suggestions have been:

- Availability and distribution of the delivery plan leaflet to all pregnant women or the plan should be included in the ANC card.
- Mass media, especially the radio and television, should be exploited in the transmission of the message on BPCR.
- Community health workers should be trained and included in providing information on BPCR in the community.

## Data Availability

The datasets used and/or analyzed during the current study available from the corresponding author on reasonable request.

## Abbreviations

ANC: Antenatal consultation
BHD: Bamenda Health District
BPCR: Birth preparedness and complication readiness
CDHS: Cameroon Demographic and Health Survey
CI: Confidence interval
CUCA: Catholic University of Central Africa
EmONC: Emergency obstetric and neonatal care
FANC: Focused antenatal consultation
MMR: Maternal mortality ratio
MPH: Ministry of Public Health
NWR: North West Region
OR: Odds Ratio
SBA: Skilled birth attendant
SDG: Sustainable Development Goal
SHS: School of Health Sciences
TBA: Traditional birth attendant
TPB: Theory of Planned Behaviour
UNFPA: United Nations Population Fund
UNICEF: United Nations Children’s Fund
WHO: World Health Organization
